# Tracking Clinic Performance to Improve Vascular Care: Minnesota Department of Health

**DOI:** 10.5888/pcd10.130301

**Published:** 2013-10-31

**Authors:** James M. Peacock

**Map:**
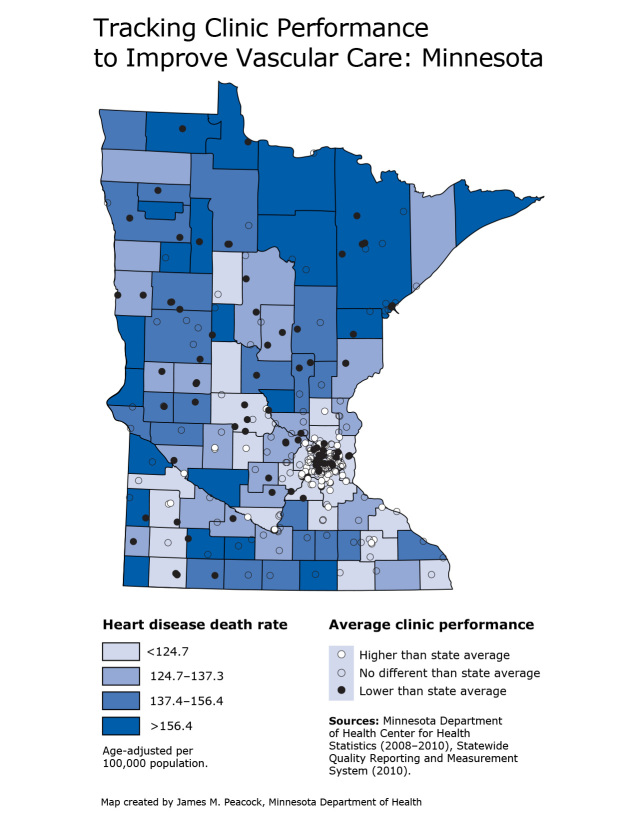
This map shows the location of physician clinics in Minnesota and their reported performance for optimal vascular care in 2010. The map also illustrates which counties experience the highest annual rates of heart disease deaths. Mapping these data together reveals that most (63%) clinics performing below the state average, excluding the Twin Cities metropolitan area, are in counties with the highest heart disease death rates. **Table:** 

Age-adjusted annual heart disease death rates per 100,000 among all people for the aggregated years 2008-2010 are mapped by county. Rates ranged from 91 to 328 per 100,000. Counties with the highest rates are primarily in northern and western Minnesota. Counties with the lowest rates are primarily in or close to the Twin Cities metropolitan area.
The measure of clinic performance on optimal vascular care indicates the percentage of vascular patients aged 18-75 years in 2010 that met all 4 of the following goals: 1) blood pressure control (ie, most recent blood pressure in the last 12 months was <130/80 mm Hg); 2) cholesterol control (ie, most recent low-density lipoprotein cholesterol <100 mg/dL); 3) daily aspirin use (or a documented contraindication); and 4) documented tobacco-free status. Clinics performing below the state average on the optimal vascular care measure are concentrated in the inner Twin Cities and in counties with the highest heart disease mortality rates.

## Background

Minnesota’s 2008 health care reform law requires physician clinics and hospitals to report annual performance on clinical quality measures to the state health department. One key measure is achieving optimal vascular care for patients with vascular disease, a form of cardiovascular disease that affects the blood vessels. Clinic performance is determined by the proportion of patients with vascular disease who meet 4 goals: appropriate aspirin therapy, blood pressure control, cholesterol control, and smoking cessation. Research has shown that achieving these goals helps reduce deaths from heart disease.

## Main Findings

Minnesota’s Heart Disease and Stroke Prevention Unit used Geographic Information Systems (GIS) to map the locations of clinics reporting on optimal vascular care, their 2010 performance on that measure, and county-level death rates for heart disease. Clinics fell into 3 performance categories: lower, no different, or higher than the state average. The map shows that outside the Twin Cities metropolitan area, most clinics performing below the state average (63%) are located in counties with the highest heart disease death rates.

## Action

The Minnesota Department of Health plans to focus improvement efforts and resources for optimal vascular care, including control of high blood pressure, on those clinics performing below the state average. In addition, these findings have led the Heart Disease and Stroke Prevention Unit to seek optimal vascular care and optimal diabetes care measure data for additional years, both at the clinic level and by the patient’s residence. Work is being undertaken to calculate heart disease mortality rates for geographic areas smaller than counties in order to assess the impact of poor control of vascular disease risk factors on heart disease mortality in Minnesota communities. In particular, the Minnesota Department of Health is interested in examining the patterns of vascular disease risk factor management and heart disease mortality among urban, suburban, small towns and rural communities throughout the state.

